# 5-Hydroxymethylcytosine discriminates between parathyroid adenoma and carcinoma

**DOI:** 10.1186/s13148-016-0197-2

**Published:** 2016-03-12

**Authors:** Elham Barazeghi, Anthony J. Gill, Stan Sidhu, Olov Norlén, Roberto Dina, F. Fausto Palazzo, Per Hellman, Peter Stålberg, Gunnar Westin

**Affiliations:** Department of Surgical Sciences, Endocrine Unit, Uppsala University, Uppsala, SE-751 85 Sweden; Department of Anatomical Pathology, Royal North Shore Hospital, Pacific Highway, St Leonards, NSW 2065 Australia; University of Sydney, Sydney, NSW 2006 Australia; Department of Surgery, Royal North Shore Hospital, Pacific Highway, St Leonards, NSW 2065 Australia; Department of Histopathology, Hammersmith Hospital, Imperial College, London, UK; Endocrine Surgery, Hammersmith Hospital, Imperial College, London, UK

**Keywords:** 5-hydroxymethylcytosine, 5hmC, Parathyroid cancer, Primary hyperparathyroidism, TET1

## Abstract

**Background:**

Primary hyperparathyroidism is characterized by enlarged parathyroid glands due to an adenoma (80–85 %) or multiglandular disease (~15 %) causing hypersecretion of parathyroid hormone (PTH) and generally hypercalcemia. Parathyroid cancer is rare (<1–5 %). The epigenetic mark 5-hydroxymethylcytosine (5hmC) is reduced in various cancers, and this may involve reduced expression of the ten-eleven translocation 1 (TET1) enzyme. Here, we have performed novel experiments to determine the 5hmC level and TET1 protein expression in 43 parathyroid adenomas (PAs) and 17 parathyroid carcinomas (PCs) from patients who had local invasion or metastases and to address a potential growth regulatory role of TET1.

**Results:**

The global 5hmC level was determined by a semi-quantitative DNA immune-dot blot assay in a smaller number of tumors. The global 5hmC level was reduced in nine PCs and 15 PAs compared to four normal tissue samples (*p* < 0.05), and it was most severely reduced in the PCs. By immunohistochemistry, all 17 PCs stained negatively for 5hmC and TET1 showed negative or variably heterogeneous staining for the majority. All 43 PAs displayed positive 5hmC staining, and a similar aberrant staining pattern of 5hmC and TET1 was seen in about half of the PAs. Western blotting analysis of two PCs and nine PAs showed variable TET1 protein expression levels. A significantly higher tumor weight was associated to PAs displaying a more severe aberrant staining pattern of 5hmC and TET1. Overexpression of TET1 in a colony forming assay inhibited parathyroid tumor cell growth.

**Conclusions:**

5hmC can discriminate between PAs and PCs. Whether 5hmC represents a novel marker for malignancy warrants further analysis in additional parathyroid tumor cohorts. The results support a growth regulatory role of TET1 in parathyroid tissue.

**Electronic supplementary material:**

The online version of this article (doi:10.1186/s13148-016-0197-2) contains supplementary material, which is available to authorized users.

## Background

Primary hyperparathyroidism (pHPT) is a common endocrine disease characterized by excessive secretion of PTH and increased level of serum calcium. Eighty to 85 % of pHPT cases are due to a benign, single adenoma and 15 % to multiglandular disease. Parathyroid carcinoma (PC) is rare, and depending on whether it is diagnosed based on pathological examination alone or biological evidence of malignant behavior, it accounts for less than 1 to <5 % of cases of pHPT [1–9]. An analysis of 286 PC cases from the USA revealed a 5-year survival rate of 86 % and a 10-year survival rate of 49 % [10]. WHO criteria for PC include demonstration of an invasive growth pattern or distant metastasis. Pathological diagnosis of PC is a challenge also because there is no widely available and completely sensitive or specific immunohistochemical marker available. Somatic inactivating mutations of CDC73/HRPT2, encoding parafibromin, are common in PCs defined by malignant behavior (approximately 70 %) and rare (<1 %) in benign disease. Negative immunohistochemical staining for parafibromin has been suggested a marker for PC by some investigators but has been reported to be less useful by others [7, 11–14]. In PC patients who had local invasion and/or metastases at initial surgery or follow-up, negative staining for parafibromin was found in 64 % and in 10 % of patients whose diagnosis was based only on classic histological features [15].

The epigenetic mark 5-hydroxymethylcytosine (5hmC) was rediscovered in mammalian cells and shown to be an intermediate in DNA demethylation of 5-methylcytosine. The ten-eleven translocation (TET) family of proteins (TET1, TET2, TET3) catalyze conversion of 5-methylcytosine to 5hmC, 5-formylcytosine, and 5-carboxylcytosine and renders passive or active DNA demethylation [16, 17]. Growing evidence suggests that 5hmC not only is an intermediate in DNA demethylation but also acts as an epigenetic mark that regulates gene expression by recruiting DNA-binding proteins [18]. 5hmC is present in many tissues and cell types and is frequently deregulated in cancer, by decreased levels of 5hmC when compared to normal tissues and some but not all tumors show changes in TET expression levels [19–28].

Here, we have for the first time determined levels of 5hmC and TET1 in PAs and PCs and investigated whether TET1 could play a role in parathyroid tumor cell growth regulation. Our study show that negative staining for 5hmC is a frequent event in PCs compared to PAs and suggest a novel potential marker for parathyroid malignancy. Furthermore, our data suggest that TET1 has a growth regulatory role in parathyroid tissue.

## Methods

### Tissue specimens

Parathyroid carcinomas (*n* = 17) from 15 patients who had local invasion and/or metastases and single parathyroid adenomas (*n* = 43) were acquired from patients diagnosed and operated on in clinical routine at the Uppsala University Hospital, Uppsala, Sweden, Department of Surgery, Royal North Shore Hospital, St Leonards, Australia, and Hammersmith Hospital, London, UK. Apparent normal parathyroid tissue (*n* = 4) was obtained as normal parathyroid gland biopsies inadvertently removed in patients subjected to parathyroidectomy. These specimens (“normals”) that stained positive for parathyroid hormone (PTH), by immunohistochemistry of frozen tissue sections, were used for comparisons with parathyroid tumor tissues. Clinical data for patients with parathyroid adenoma or carcinoma are shown in Additional file 1: Table S1 and Additional file 2: Table S2, respectively. Informed consent and approval of the Uppsala Local Ethical Committee, the Northern Sydney Local Health District Human Research Ethics Committee, and by the Imperial College Research Ethics Committee, London, were achieved.

### DNA sampling and dot blot analysis

Genomic DNA was extracted from frozen surgical specimens or cultured cells using DNeasy Blood and tissue kit (Qiagen GmbH, Hilden, Germany) and paraffin-embedded tissue sections using QIAamp DNA FFPE tissue kit (Qiagen GmbH) according to manufacturer’s instructions. 5hmC DNA standard (Zymo Research Corporation, Irvine, CA, USA) was used as a control. One microgram DNA was denatured in 0.1 M NaOH at 95 °C for 10 min, then placed on ice and neutralized with 1 M ammonium acetate. Twofold serial dilutions of the DNA samples were prepared and spotted onto Hybond-N+ nylon membrane (GE Healthcare, Piscataway, NJ, USA) in a Bio-Dot apparatus (Bio-Rad Laboratories, Inc., Hercules, CA, USA). The spotted membrane was fixed with UV irradiation (GS Gene Linker UV chamber, Bio-Rad), blocked with 5 % skimmed milk, and incubated overnight with a rabbit polyclonal anti-5hmC antibody [19, 25, 29] (1:10K dilution, 39791; Active Motif, Carlsbad, CA, USA) and then appropriate HRP-conjugated secondary antibody. Signals were visualized with the enhanced chemiluminescence system (GE Healthcare). The same membrane was stained with 0.02 % methylene blue in 0.3 M sodium acetate to ensure equal spotting of the total DNA on the membrane. The second dot blot signal from the top for each serial dilution was used to quantify intensities by the NIH Image-J software according to the program’s instructions [30] and the 5hmC relative intensity was calculated by dividing the value for each sample by the value of the standard. A linear relationship was obtained for the serially diluted 5hmC standard (not shown).

### Immunostaining

Immunohistochemistry: Paraffin embedded tissue sections were deparaffinized with xylene and rehydrated through descending alcohol concentrations and distilled water. Sections were treated with 3 % hydrogen peroxide and heated in EDTA pH 8.0 (Life Technologies Corporation, Carlsbad, CA, USA ), for 40 min with microwave at 300-W power. The sections then were incubated with 2 M HCl for 2.5 min and treated with normal goat serum and the rabbit polyclonal anti-5hmC antibody (1:6000 dilution, Active Motif), rabbit polyclonal anti-TET1 (HPA019032; Prestige Antibodies, Sigma-Aldrich Sweden AB, Stockholm, Sweden) or normal horse serum and goat polyclonal anti-PTH antibody (sc-9678; Santa Cruz Biotechnology, Santa Cruz, CA, USA). The sections were washed three times with PBS, then incubated with a proper secondary antibody and ABC complex. DAB was used for visualization. Frozen tissue sections (apparent normal parathyroid tissue and parathyroid carcinoma #12 and #13) were first fixed in formalin and then stained as described above but excluding incubation in 2 M HCl. Immunofluorescence: The sections were treated and incubated with rabbit polyclonal anti-5hmC antibody (1:6000 dilution, Active Motif) as mentioned above, then washed three times with PBS (0.05 % Tween20) and incubated with proper fluorescence secondary antibody (Alexa 594, Life Technologies). Sections were washed again and mounted with Vectashield with Dapi (Vector Laboratories, Inc., Burlingame, CA, USA). Normal liver tissue was used as positive control for TET1.

### Western blotting

Protein extracts prepared using Cytobuster Protein Extract Reagent (Merck Millipore, Billerica, MA, USA) with complete protease inhibitor cocktail (Roche Diagnostics Scandinavia AB, Bromma, Sweden). Rabbit polyclonal anti-TET1 (GTX124207; GeneTex Inc, Irvine, CA, USA) and goat polyclonal anti-Actin (sc-1616; Santa Cruz Biotechnology) were used. After incubation with the appropriate secondary antibody, bands were visualized using the enhanced chemiluminescence system (GE Healthcare).

### Colony formation assay and measurement of apoptosis

sHPT-1 parathyroid tumor cells [31] were distributed onto 35-mm dishes (2 × 10^5^) in DMEM/10 % fetal bovine serum (Sigma) and transfected in triplicates with 1 μg pIRES-hrGFP ll-TET1-FL [32] or empty vector (pIRES-hrGFP ll) using FuGENE 6 transfection reagent (Promega Biotech AB, Nacka, Sweden), according to manufacturer’s instructions. Twenty four hours after transfection, 2000 sHPT-1 cells were distributed onto six-well plates in triplicates. After additional 24 h, a fresh medium with 0.2 mg/ml Neomycin (G418, Sigma-Aldrich) was added and refreshed every 72 h. After 10-day selection in Neomycin, the cells were fixed with 10 % acetic acid/10 % methanol and stained with 0.4 % crystal violet, and visible colonies were counted. HEK293T cells (1.5 × 10^6^) were transfected with FuGENE 6 transfection reagent (Promega Biotech AB). After 24 h, 8000 HEK293T cells were distributed onto six-well plates in triplicates and the next day 0.2 mg/ml Neomycin was added to culture medium. After 10 days in selection cells were fixed, stained and counted as described above. Successful transfection was determined by real-time quantitative RT-PCR and western blotting for sHPT-1 and HEK293T, after 72 h. The human parathyroid tumor cell line sHPT-1 was used at cell passages 18–30, and the cells were routinely tested for PTH expression by immunostaining of cultured fixed cells [31]. The human embryonic kidney cell line HEK293T was obtained from ATCC (LGC, Promochem, Sweden) and used at cell passages 10–30. Apoptosis was measured in sHPT-1 cells 72 h after transfection and after 10 days of antibiotic selection, using the Cell Death Detection ELISA kit (Roche Molecular Biochemicals, Mannheim, Germany), according to the manufacturer’s instructions. As a positive control, cells were incubated with 0.1 μg/ml camptothecin (Sigma-Aldrich) for 72 h. Transfected cells were also incubated with FITC-labeled annexin V and propidium iodide (Sigma-Aldrich) and analyzed by flow cytometry on a FACS Canto ІІ (BD Biosciences). Annexin V-FITC binds to phosphatidylserine translocated to the external portion of the membrane as a marker of apoptosis, and propidium iodide distinguishes dead cells with ruptured membrane.

### Real-time quantitative PCR

 DNA-free total RNA was extracted using RNeasy Plus Mini kit (Qiagen GmbH) according to manufacturer’s instructions and successful treatment with DNase I of all RNA preparations using TURBO DNA-free™ kit (Life Technologies Corporation) was established by PCR analysis. After reverse transcription of RNA preparations with random hexamer primers using First-strand complementary DNA (cDNA) synthesis kit (GE Healthcare), real-time quantitative RT-PCR was performed on StepOnePlus RealTime PCR systems (Life Technologies Corporation) using TaqMan gene expression Master Mix and assays for TET1 transcript (Hs00286756_m1) and GAPDH (Hs02758991_g1). Each cDNA sample was analyzed in triplicate.

### Statistical analysis

ANOVA test was used to calculate differences in relative 5hmC level between the three biological groups and to compare clinical data between the four groups of adenomas. Bonferroni test was used to adjust the *p* values (due to having a few number of samples, Kruskal-Wallis test was used to check the significance of the *p* values obtained from the ANOVA test). Differences in colony formation assay and real-time quantitative RT-PCR was evaluated using unpaired *t* test, and data are presented as mean ± SEM. Statistical analysis was performed using R version 3.1.1 (2014-07-10). *p* < 0.05 was considered significant.

## Results

### Reduced global level of 5-hydroxymethylcytosine in parathyroid tumors

**Fig. 1:**
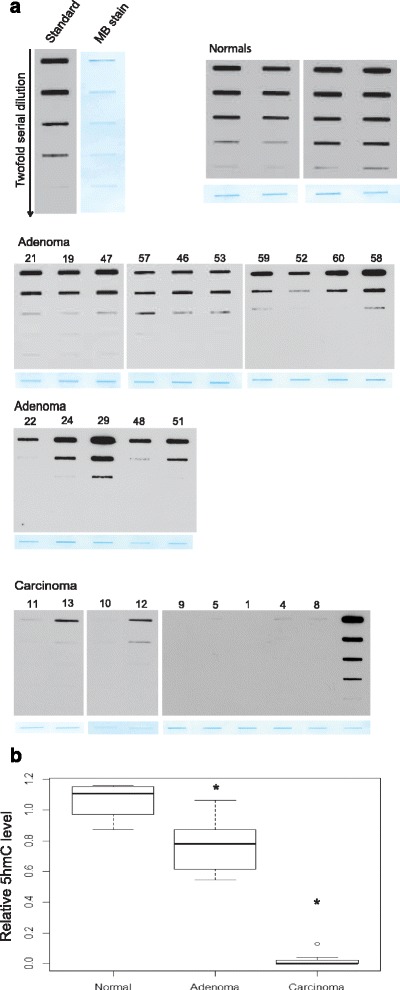
DNA immune-dot blot assay of 5hmC using a rabbit polyclonal anti-5hmC antibody. **a** Analysis of twofold serial dilutions of DNA. Standard (control 5hmC-DNA), normal parathyroid tissue specimens (*n* = 4), PAs (*n* = 15), and PCs (*n* = 9). *MB* methylene blue loading control represents the least diluted DNA sample. **b** Quantification of the results in **a**. *p < 0.05

A DNA immune-dot blot assay was established using a previously validated rabbit polyclonal anti-5hmC antibody [19, 25, 29]. Comparisons of twofold serial dilutions of total DNA isolated from PCs (*n* = 9), PAs (*n* = 15), and apparent normal parathyroid biopsies (*n* = 4) revealed a significantly (*p* < 0.05) reduced level of 5hmC in the adenomas and the carcinomas compared to the normal tissue samples (Fig. 1a, b). The global 5hmC level was most severely reduced in the PCs.

### Immunohistochemistry of 5hmC discriminates between adenoma and carcinoma

**Fig. 2:**
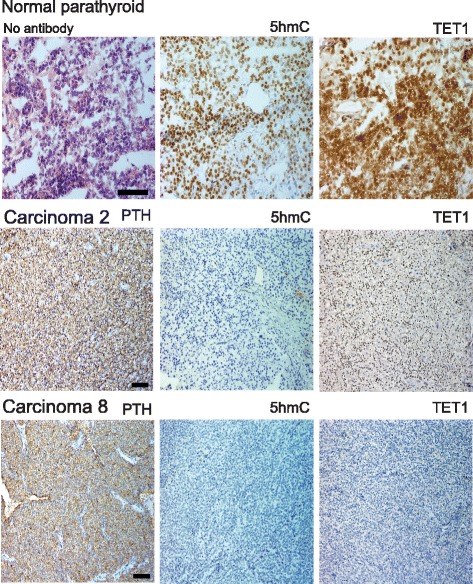
Immunohistochemical analysis of a normal parathyroid tissue specimen (frozen) and paraffin imbedded PCs (*n* = 2). *Top left*, staining without primary antibody. Same numbering of the PCs as in Additional file 3: Table S3. Both carcinomas stained negative for 5hmC, carcinoma 2 positive for TET1, and carcinoma 8 negative for TET1. *Scale bars*, 50 μm (normal parathyroid) and 100 μm (PCs). Staining results of additional PCs are shown in Additional file 4: Figure S1

Additional PCs were available for immunohistochemical analysis. In comparison to normal parathyroid cells that stained positively, all 17 analyzed carcinomas from 15 patients stained negatively for 5hmC (Fig. 2 and Additional file 3: Table S3, denoted, − negative, undetectable). These results are in line with those obtained from the DNA immune-dot blot assay, where the global 5hmC level was very low to almost undetectable in the analyzed PCs (Fig. 1a, b).

**Fig. 3:**
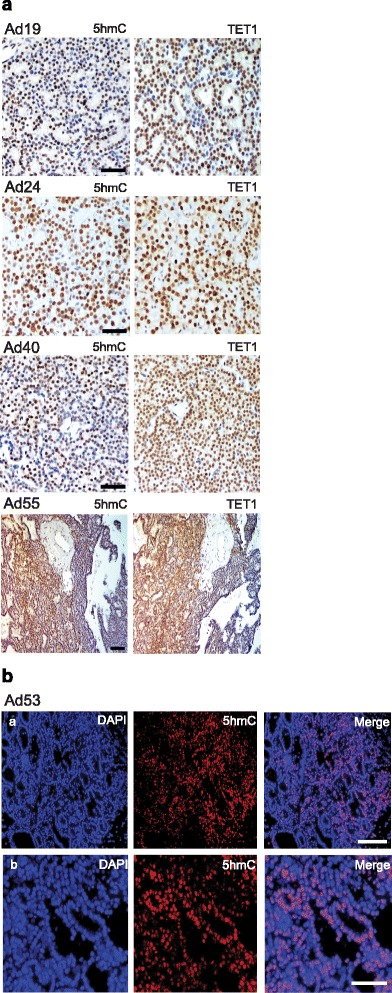
**a** Representative results from immunohistochemical analysis of 5hmC and TET1 in PAs (*n* = 4). Ad19 shows mosaic staining (M) for both (a mixture of positive and negative cells), Ad24 shows + (almost all cells stained positive regardless of strength), Ad40 shows M for 5hmC and + for TET1, and Ad55 shows VH (variable heterogeneous, areas with positive staining together with areas of negative staining). *Scale bars*, 50 μm (Ad 19, Ad24, Ad40) and 100 μm (Ad55). **b** Immunofluorescence was applied to demonstrate typical appearance of mosaic staining (M) of 5hmC at two magnifications: *a* scale bar 100 μm; *b* scale bar 50 μm

In contrast, the observed staining patterns of the adenomas were generally heterogeneous with different appearances and these were denoted with M (mosaic, a mixture of positive and negative cells); VH (variable heterogeneous, areas with positive staining together with areas of negative staining, and + (almost all cells stained positive regardless of strength). The results are summarized in Additional file 3: Table S4-S7 and representative staining patterns are shown in Fig. 3a, b. Overall, 7 adenomas showed positive staining for almost all cells (+, Additional file 3: Table S4) and 36 PAs stained positively for 5hmC but with the aberrant variable appearance described above. Of the 36 PAs with aberrant variable appearance of 5hmC, 23 adenomas showed mosaic staining (M) with a mixture of positive and negative cells (Additional file 3: Table S4–S6), 6 adenomas showed mosaic staining (M) together with variable heterogeneity (VH), areas of positive staining together with areas of negative staining (Additional file 3: Table S6), and 7 adenomas showed VH only (Additional file 3: Table S7). The presence of 5hmC negatively staining cells in PAs is consistent with the observed reduction of the global 5hmC level by the DNA immune-dot blot assay (Fig. 1a, b). Also, three PAs (#22, 24, 29) that stained positive for almost all cells regardless of strength (+, Additional file 3: Table S4) showed reduced global 5hmC level (Fig. 1a).

To summarize, all 17 PCs showed negative/undetectable staining for 5hmC and all 43 PAs contained 5hmC positive cells. 5hmC may therefore be used as a marker to discriminate between benign and malignant parathyroid tumors.

### TET1 expression and 5hmC level establish groups of PAs and relate to tumor weight

**Fig. 4:**
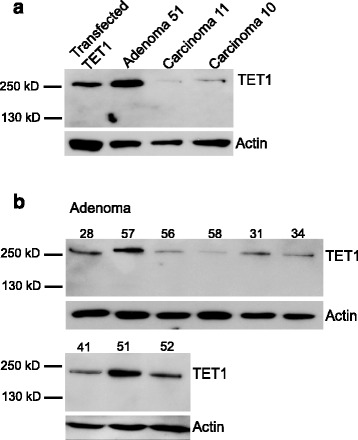
Western blotting analysis of TET1. **a** Transfected TET1 (sHPT-1 cells), PA51, PC11, and PC 10. **b** PAs (*n* = 9)

**Fig. 5:**
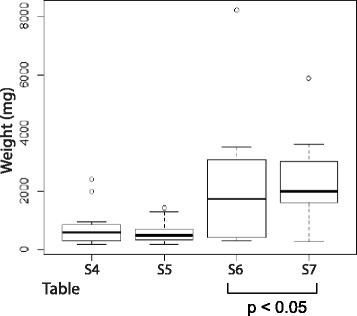
Association of PAs of Additional file 3: Table S6 and S7 to tumor weight (and not to serum PTH, serum calcium or age). These PAs showed a more severe aberrant staining pattern of 5hmC and TET1 (M, VH). Clinical data is presented in Additional file 1: Table S1

TET protein family members are responsible for the conversion of 5mC to 5hmC (16) and altered 5hmC levels may be caused by deregulated TET expression [26–28]. We therefore performed immunohistochemical analysis of TET1 in our cohort of PAs and PCs (Additional file 3: Table S3–S7, Figs. 2 and 3a). The observed staining patterns of TET1 resembled those of 5hmC. TET1 expression was undetectable in 7 out of the 17 PCs (Additional file 3: Table S3), variable heterogeneous (VH), with areas of positive staining together with areas of negative staining, was seen in 6 carcinomas, and 4 carcinomas showed positive staining in most cells, regardless of strength (+). A primary PC and metastasis from the same patient were available for analysis and showed the same staining patterns of 5hmC and TET1 (Additional file 3: Table S3, tumor nos. 14 and 15). The staining for TET1 in the PCs and PAs was nuclear or nuclear/cytoplasmic, with no associations to overall staining patterns (+, M, VH, data not shown). Staining for parafibromin, encoded by HRPT2, was available for PC nos. 1–9. No associations of negative or positive parafibromin staining to TET1 expression (−, +, VH) were observed (Additional file 2: Table S2 and Additional file 3: Table S3). The staining results for 5hmC and TET1 were used to group the analyzed PAs as presented in Additional file 3: Tables S4–S7. Additional file 3: Table S4 presents tumors with analogous mosaic staining (M) of both 5hmC and TET1 and tumors with positive staining of 5hmC/TET1 for almost all cells regardless of strength (+). Additional file 3: Table S5 contains PAs with mosaic (M) appearance of 5hmC and positive staining of TET1 for almost all cells regardless of strength (+). Additional file 3: Tables S6 and S7 present adenomas with more severe aberrant staining patterns. Mosaic (M) staining for 5hmC together with VH (variable heterogeneous, with areas of positive staining together with areas of negative staining, Additional file 3: Table S6) or analogous VH appearance for 5hmC and TET1 (Additional file 3: Table S7). All PAs showed positive TET1 staining but with a similar aberrant staining pattern (M, VH) of TET1 and 5hmC in about half of the PAs. PTH expression was evident also in areas of negative 5hmC and TET1 staining (data not shown). Western blotting analysis was also done for TET1 and showed very low expression levels in the two analyzed PCs (Fig. 4a) and low to variable expression levels in the nine analyzed adenomas (Fig. 4b). When clinical parameters (Additional file 1: Table S1) of the patients (serum PTH, serum calcium, tumor weight, age) were related to the staining results of the different PA groups (Additional file 3: Table S4-S7), PAs of Additional file 3: Table S6 and S7 was found to be associated with a higher tumor weight (Fig. 5). Thus, a more severe aberrant staining pattern of 5hmC and TET1 associated to tumor mass and may therefore interfere with the tumor cell growth regulatory control.

### A growth regulatory role of TET1 in parathyroid cells

**Fig. 6:**
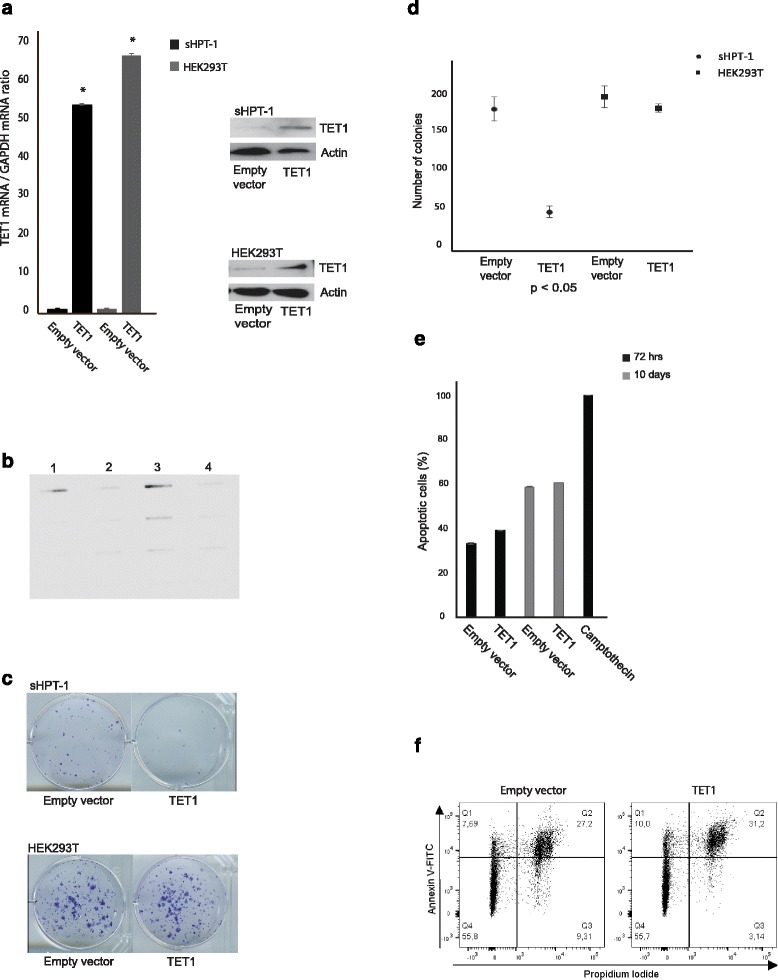
Colony formation assay in the parathyroid cell line sHPT-1 and HEK293T cells. **a** Transfection of a TET1 expression vector and not the empty vector resulted in increased expression of TET1 at the messenger RNA (mRNA) and protein levels and **b** increased the global level of 5hmC (lanes 1 and 3, transfection of sHPT-1 cells for 72 h and 10 days, respectively; lanes 2 and 4, empty vector). **c** Colony formation assay, representative results are shown. Transfected cells were selected by incubation with G418 (neomycin) for 10 days. sHPT-1 cells also appeared as smaller colonies not necessarily apparent in **c**. **d** Quantification of triplicates (mean ± SEM); sHPT-1/TET1 = 46 ± 7.1 and sHPT-1/empty vector = 175 ± 14.9. HEK293T/TET1 = 176 ± 5.6 and HEK293T/empty vector = 190.6 ± 13.7. **e** Effects on apoptosis was analyzed in triplicates by quantifying cytoplasmic histone-associated-DNA-fragments 72 h after transfection and after 10 days of G418 selection. Incubation in 0.1 μg/ml camptothecin for 72 h was used as a positive control. **f** Flow cytometry analysis of sHPT-1 cells 72 h after transfection and after staining with annexin V-FITC and propidium iodide. Early apoptotic cell population in the upper *left quadrant* and late apoptotic cells in the *upper right quadrant*. Alive and dead cell populations in the *lower left and right quadrant*, respectively. Incubation in 0.1 μg/ml camptothecin for 4 h was used as a positive control (data not shown)

In order to test experimentally whether TET1 could interfere with parathyroid cellular growth, the parathyroid tumor cell line sHPT-1 and also HEK293T cells were transfected with a TET1 expression vector or empty vector in a colony formation assay. Increased expression of TET1 and increased level of 5hmC were detected after transfection (Fig. 6a, b), and this resulted in a significant reduced number and also size of sHPT-1 cell colonies (Fig. 6c, d). This was caused by reduced growth capability as no effect on apoptosis was detected after transfection of TET1 to sHPT-1 cells, as determined by quantifying cytoplasmic histone-associated-DNA-fragments and by flow cytometry of cells stained with annexin V-FITC and propidium iodide (Fig. 6e, f). No growth inhibitory effect was seen in HEK293T cells (Fig. 6c, d). Overall, these results strongly support a growth regulatory role of TET1 in parathyroid tumor cells.

## Discussion

PC is difficult to diagnose; it has been reported that up to half of metastatic or recurrent PCs were first diagnosed as benign [33]. Correct diagnosis may suggest aggressive surgery to decrease the risk of recurrence. Several immunohistochemical markers of parathyroid malignancy have been suggested (e.g., APC, galectin-3, parafibromin, PGP9.5, Rb), but none has been widely accepted as highly sensitive and specific [7, 9]. Our novel results demonstrated that all 17 analyzed PCs stained negatively for 5hmC. In contrast, all 43 adenomas showed positive staining for 5hmC. Thus, it seems that 5hmC can discriminate between PAs and PCs. We suggest that negative staining for 5hmC may present a novel potential marker for parathyroid malignancy and that this warrants investigations in additional cohorts of parathyroid tumors from patients with pHPT.

TET1 was first identified as a fusion partner of the mixed lineage leukemia (*MLL*) gene in acute myeloid leukemia, and it is now clear that TET1 plays an essential oncogenic role in MLL-rearranged leukemia [27, 28, 34]. On the contrary, in many solid tumors, including breast, lung, prostate, and colorectal, TET1 expression is down-regulated with reduced levels of 5hmC, and TET1 mutations are rarely observed [22, 26–28]. TET1 has been shown to play a role as tumor suppressor in breast, prostate, and colon cancers [35–37].

We found that expression of TET1 was undetectable in 7 out of the 17 PCs (41 %) and in 6 PCs the expression of TET1 was heterogeneous with areas of positive cells together with areas of negative cells. Since a clear correlation between decreased 5hmC levels and TET1 expression was observed only in 41 % of the PCs, other mechanisms must be involved. Besides indirect mechanisms, it is possible that TET2 also contributes to the observed reduced levels of 5hmC in some of the analyzed tumors [26, 28]. This will be addressed in future experiments when an appropriate antibody to TET2 is available.

A significantly higher tumor weight was found associated to PAs that displayed a more severe aberrant staining pattern of 5hmC and TET1. This strongly suggests a growth regulatory role of 5hmC and TET1. Consistently, overexpression of TET1 resulted in inhibition of parathyroid tumor cell growth. These observations support a possible role of TET1 as a tumor suppressor gene in this tissue.

Reduced expression of TET1 is expected to result in direct deregulation of parathyroid target genes, as for example described in colon cancer for the DKK3 and DKK4 inhibitors of the WNT pathway [37]. Interestingly, impaired expression of another inhibitor of the WNT signaling pathway by DNA methylation, the tumor suppressor protein APC (adenomatous polyposis coli), has been associated with PC malignancy [38, 39].

## Conclusions

PC is very rare, and in the absence of disseminated disease, it is sometimes difficult to distinguish PC from benign adenoma. At present, a widely available and sensitive or specific immunohistochemical marker for PC is not available. Here, we have analyzed a relatively large number of PCs and found 5hmC to present a new potential negative marker for parathyroid malignancy. Our novel findings also include an association of a more aberrant immunohistochemical staining pattern of 5hmC and TET1 to tumor weight for the PAs and a direct demonstration in vitro of a growth regulatory role for TET1 in parathyroid tumor cells.

## Additional files

Additional file 1: Table S1. Clinical data (serum PTH, serum calcium, tumor weight, age) for patients with parathyroid adenoma. (EPS 3563 kb) Additional file 2: Table S2. Clinical data for patients with parathyroid carcinoma. Tumor nos. 1–9, 13, 14, 16, and 17 present primary PCs and 10–12 and 15 metastases. Tumor nos. 10 and 11 and 14 and 15 were from the same patient, respectively. The following five PCs have been published previously. Tumor no. 3 [13], no. 4 [40], and no. 11–13 [41]. Negative staining for parafibromin was detected in PC nos. 2, 3, and 5–8 and positive staining in nos. 1, 4, and 9 (unpublished results). (EPS 1549 kb) Additional file 3: Tables S3–S7.
**Table S3.** IHC results for carcinomas. **Table S4–S7.** IHC results for adenomas. (EPS 3060 kb) Additional file 4: Figure S1. A, Immunohistochemical analysis of 5hmC and TET1 in paraffin imbedded PCs. Scale bar, 100 μm. All PCs stained positive for PTH (not shown). Same numbering of the PCs as in Additional file 3: Table S3. Carcinoma nos. 2 and 8 are shown in Fig. 2. B, Normal liver tissue was used as positive control and stained with or without the primary TET1 antibody. Clear TET1 nuclear staining was observed only in the presence of the primary antibody. Scale bar, 100 μm. (EPS 324013 kb)
